# Supplementary figures and images for: Identification of Protein–Phenol Adducts in Meat Proteins: A Molecular Probe Technology Study
Source: Foods. 2023 Nov 23;12(23):4225. doi: 10.3390/foods12234225 (PMC10706583; doi:10.3390/foods12234225)

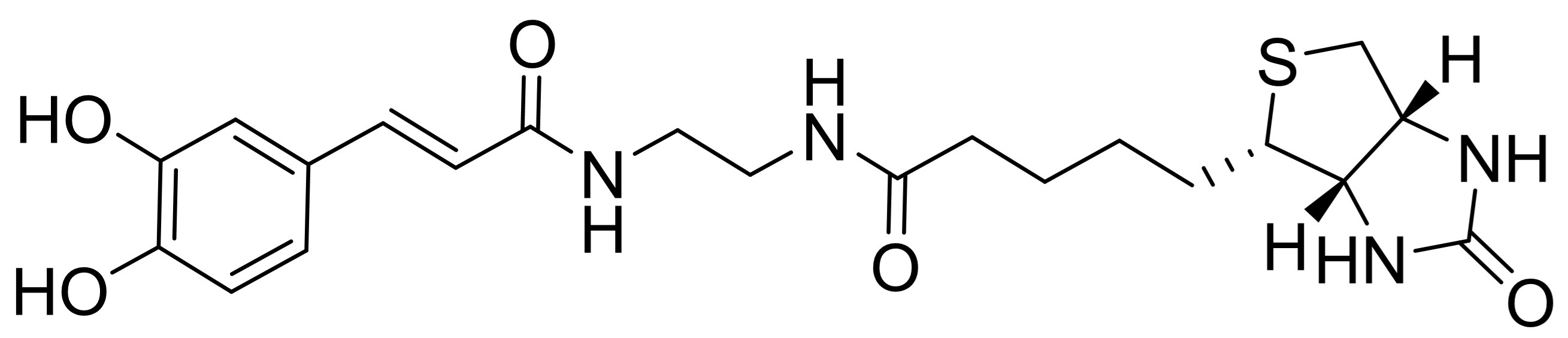

Supplement: Supplementary file 1 [file foods-12-04225-s001.zip › Figure S1.tif]

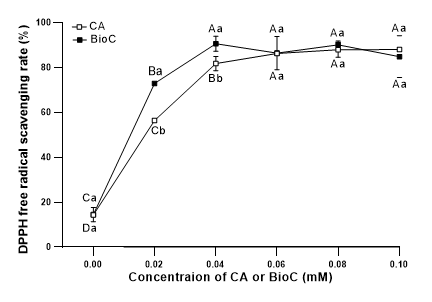

Supplement: Supplementary file 1 [file foods-12-04225-s001.zip › Figure S2.tif]
